# A Combination of Lactic Acid Bacteria and Molasses Improves Fermentation Quality, Chemical Composition, Physicochemical Structure, *in vitro* Degradability and Rumen Microbiota Colonization of Rice Straw

**DOI:** 10.3389/fvets.2022.900764

**Published:** 2022-06-08

**Authors:** Xu Chen, Yulin Ma, Muhammad Zahoor Khan, Jianxin Xiao, Gibson Maswayi Alugongo, Shengli Li, Yajing Wang, Zhijun Cao

**Affiliations:** ^1^State Key Laboratory of Animal Nutrition, College of Animal Science and Technology, China Agricultural University, Beijing, China; ^2^Department of Animal Sciences, Faculty of Veterinary and Animal Sciences, University of Agriculture, Dera Ismail Khan, Pakistan

**Keywords:** lactic acid bacteria, molasses, rumen microbial colonization, rice straw, *in vitro* digestibility

## Abstract

**Aims:**

This study aims to evaluate the effect of lactic acid bacteria (LAB) and LAB-molasses (LAB + M) combination on the fermentation quality, chemical composition, physicochemical properties, *in vitro* degradability of rice straw and the characteristics of rumen microbial colonization on rice straw surface.

**Methods and Results:**

There were three pretreatments, including control (not treated, Con), treated with LAB, or LAB + M. The results showed that both LAB and LAB + M treatments altered the physical and chemical structures of rice straw and were revealed by scanning electron microscopy (SEM) and X-ray diffraction analysis (XRD) spectroscopy, respectively. Moreover, both LAB and LAB + M pretreated rice straw increased the crude protein (CP) content, dry matter (DM) recovery, and *in vitro* digestibility and decreased the pH value, neutral detergent fiber (NDF), and acid detergent fiber (ADF) contents. The LAB + M pretreated rice straw increased the gas production (GP72) and rumen microbial colonization on the rice straw surface.

**Conclusions:**

It is observed that LAB + M treatment could increase digestibility and the rumen microbial colonization on the rice straw surface. Therefore, LAB + M treatment can provide an alternative strategy to improve the quality of rice straw. Significance and impact of the study: This study provides an optimal pretreatment to improve the rice straw digestibility and rumen microbial colonization.

## Introduction

Rice straw is one of the most abundant crop residues in the world, especially in China. It is commonly used as the source of roughage in ruminants' nutrition due to its richness and low cost. However, the recalcitrant cellulose—hemicellulose—lignin structure protects the rice straw biomass from the access of microorganisms (1), results in low degradation during ruminal fermentation, and subsequently limits intake in animals (2). Increasing the degradation of rice straw would help to increase the utilization of this abundant agricultural by-product in ruminants and hence reduce the status of shortage of high-quality forage.

The lactic acid bacteria (LAB) plays a role in the preservation and fermentation of forage crops within inoculated silages (3). LAB help to suppress the growth of spoilage microorganisms by reducing pH levels in fermented forages (4). Previous studies have shown that exogenous LAB can improve the quality of rice straw (5) and grass (6) silages. However, the insufficient contents of water-soluble carbohydrates (WSCs) in raw rice straw may lead to the accumulation of low lactic acid and increased pH value, allowing undesirable microorganisms such as *clostridia* to grow in the silage (7). Therefore, it might be helpful to add cheap sources of exogenous WSC, such as molasses (8). Sufficient WSC not only provides sufficient substrates for LAB fermentation but also increases the nutritional quality of rice straw silage.

Ruminal microorganisms are the most dominant domain contributing to the digestion and conversion of feedstuff to volatile fatty acids and microbial proteins (9). The attachment of microorganisms to the consumed forage is a key step in the forage degradation process and affects the degradation of the dietary (10). However, many studies have mainly focused on the changes in the colonization of microorganisms in the rumen degrading process of roughage that has not been pretreated. For example, Huws et al. reported that colonization of bacteria in the freshly ingested perennial ryegrass (11). However, not a single study has reported the rumen microbial colonization after pretreatment of roughage raw materials. Moreover, the physical structure and chemical characteristics of the feed particles are the key factors that affect microbial colonization (12). Therefore, further research on the changes in microbial colonization and the digestion of roughage after pretreatment is very important for the improvement in the efficiency of feed nutrient utilization by ruminants.

In this study, LAB or LAB + M pretreated rice straw with respect to its fermentation quality, chemical composition, physicochemical properties, *in vitro* degradability of rice straw, and the characteristics of rumen microbial colonization on rice straw surface.

## Materials and Methods

All animal procedures used in this study were reviewed and approved by the Animal Care Committee of the College of Animal Science and Technology of China Agriculture University (protocol number: 2013-5-LZ).

### Substrate and Treatment

Rice straw was collected from suburban farmland in Henan province, China. Mature whole rice plant was thrashed to remove the grain and the rice straw was sun-cured. The rice straw was cut into 3–5 cm using a guillotine cutter. The LAB and LAB + M were evenly sprayed on the rice straw, as per their respective treatments. For each treatment, the amounts of molasses and water were calculated in advance and weighed, whereas materials were separately added into the distilled water to adjust the moisture content to 70%.

The LAB were (1) a mixture of both homofermentative and heterofermentative LAB inoculants that were consisted of three strains of *Lactobacillus (L. plantarum* PS-8, *L. plantarum* PS-F, and *L. buchneri* HM-01), whose details have been described previously (2, 13) molasses (purchased from the Hebei Shuntong Encyclopedia Trading Co., Ltd., China). The three treatments were as follows: rice straw treated without (control, Con; supplemented with distilled water to adjust the moisture content to 70%) or with LAB (added at 1 × 10^11^ CFU/g of fresh material) or LAB + M group (mixture of molasses and LAB, molasses was added to 3% of the fresh material). The rice straw was ensiled in triplicate for each treatment in laboratory polyethylene bags (25 cm by 35 cm; Beijing Shengya Yuda Biological Technology Co., Ltd, Beijing, China) and sealed by a food vacuum sealing machine (Konka KZ-ZK007; Dongguan Yijian Packaging Machinery Co. Ltd, Dongguan, China). All forages were ensiled for 7, 15, 30, and 45 days before opening.

### Chemical Composition and *in vitro* Digestibility Analyses

Dry matter and crude protein contents were determined according to AOAC methods (14). The weight and DM content before and after ensiling were weighed to calculate the rate of DM loss, whereas NDF and ADF were determined using the procedures adopted by Van Soest et al. (15).

Rumen fluids from three healthy Holstein cows were collected 2 h after the morning feeding, mixed, and placed in a 39°C pretemperature vacuum flask and then immediately transported to the laboratory. Cows were fed a TMR containing 5.6% oat hay, 11.5% alfalfa hay, 8.3% alfalfa silage, 24.5% corn silage, and 50.1% concentrate (13.7% of stem-flaked corn, 5.0% of corn, 8.4% of soybean meal, 5.2% of soybean hull, 4.4% of corn DDGS, 3.3% of sprayed corn skin, 3.3% of cottonseed meal, 2.9% of molasses, 0.5% of Berg + Schmidt, 0.3% of XP XPC, 2.4% of premix, 0.4% of NaHPO_3_, and 0.3% OPTIGEN of DM) which offered three times a day (07:00, 14:00, and 18.30). Cows had free access to water throughout the day.

The *in vitro* gas production was determined using an automated trace gas recording system (AGRS) for microbial fermentation, as described previously (16). Briefly, 500 mg (DM basis) of representative samples (rice straw samples had been fermented for 45 days) (6 replicates) of each treatment group was weighed and placed in 120-ml glass bottles. About 50-ml of a freshly prepared buffer solution was added to each bottle (17). All bottles were purged with anaerobic N_2_ for 5 s, sealed with rubber plug and Hungate screw caps, and individually connected with medical plastic infusion pipes to the AGRS, according to the procedure introduced by Zhang and Yang (18). All the bottles were incubated at 39°C for 72 h, and each batch culture system run had 6 bottles of samples for blank correction. The bottles were removed from the AGRS system after 48 h of incubation. The pH value of the culture medium was immediately determined. The *in vitro* digestibility of DM (IVDMD), NDF (NDFD), and ADF (ADFD) was calculated using differential subtraction according to the DM content of substrates before and after *in vitro* incubation.

### Structural Analyses

The morphological and structural images of the rice straw were obtained by emission scanning electron microscopy (SEM, ASU 3500, Japan) according to the manufacturer's instructions. Briefly, morphological changes in biomass before and after pretreatment with additives were observed at a magnification of 1,500. Prior to imaging, the samples (rice straw samples had been fermented for 45 days) were sputter-coated with platinum to make them electrically conductive.

The X-ray diffraction (XRD) method was applied for the detection of cellulose crystallinity index (CrI) of samples (rice straw samples had been fermented for 45 days) as described by Zhang et al. (19). The XRD was conducted with a Siemens D-5000 diffractometer (Bruker, Ettlingen, Germany), and Cu-K radiation was generated at 40 kV and 20 mA. Samples were scanned from 3° to 40° with a step size of 0.02 and 3 s per step. The cellulose crystallinity index (CrI) was calculated using the following formula (20):


$$\begin{array}{l} CrI=\left(I_{002}-I_{\text{am}}\right)/I_{002} \end{array}$$

where I_002_ is the scattered intensity at the main peak for cellulose type I and I_am_ is the scattered intensity due to the amorphous portion evaluated as the minimum intensity between the main and secondary peaks.

### *In situ* Rumen Incubation

The experimental procedures of *in situ* rumen incubation were done according to method exercised by Gharechahi et al. (21). In brief, rice straw air-dried samples that had been fermented for 45 days were ground into a 2-mm sieve using a Wiley mill (KRT-34; KunJie, Beijing, China). About 5 g of the milled samples was weighed and placed in nylon bags (8 × 16 cm; pore size = 45 mm) in six replications. These bags were incubated for 0.5, 4, 12, and 24 h in the rumen of three fistulas cows (two replications per cow). After removing from the rumen, the bags were washed with sterile saline to remove loosely attached microbiota and then immediately frozen on dry ice and transported to the laboratory for storage at −80°C until DNA extraction.

### DNA Extraction and Quantitative Real-Time PCR Analysis

Total microbial genomic DNAs were extracted from 200 mg of the rumen-incubated rice straw sample. The DNA was extracted according to the protocol for pathogen detection in stool using an EZNA Stool DNA kit (Omega Biotek, Norcross, GA, US). For bacteria, the V3-V4 variable region of the 16S rDNA was targeted using primers Eub338F (ACTCCTACGGGAGGCAGCAG) and Eub806R (GGACTACHVGGGTWTCTAAT). Quantitative real-time PCR (q-PCR) was performed according to the procedures described by Jiao et al. (22). A standard curve was generated using plasmid DNA containing the exact 16S/18S rRNA gene inserts, and the standard curve met the following requirements: *R*^2^ > 0.99. The qPCR assay was performed to generate fragments of 460 base pairs (bp) suitable for paired-end sequencing on the Illumina Miseq system (Shanghai Majorbio Bio-pharm Technology Co., Ltd). The reactions were performed in a 20 μl mixture containing 10 μl of 2X Taq Plus Master Mix, 0.8 μl of each primer (5 μM), 7.4% of ddH_2_O, and 1 μl of each reaction which was used as a template of PCR. Each sample was performed in triplicate for PCRs.

### Data Analysis

The cumulative gas production (GP_72_) (mL/g) data were recorded using the AGRS system and fitted to the Groot model as per Equation (1) (23).


(1) $$\begin{array}{r} GP_{\text{t}}=A/\left[1+{\left(C/t\right)}^{\text{B}}\right] \end{array}$$

“A” is the asymptotic gas production (ml/g); “B” is a sharpness parameter determining the curve's shape; “C” is the time (h) at which half of A is reached; and “t” is *in vitro* incubation time (h).

The results of the 6 bottles per treatment within each run were averaged and then analyzed using a mixed model with SPSS 24.0 (SPSS Inc., Chicago, IL). The two-way ANOVA analysis was performed to examine the effect of treatment with treated groups and the ensiling period on the chemical composition of rice straw. Duncan's multiple comparison method was carried out to compare the differences between the means; *p* <0.05 was used to show the significance levels. The DNA sequencing data were analyzed on a free online platform of Majorbio tools https://cloud.majorbio.com/page/project/p.html.

## Results

### Physical Structure and Physicochemical Properties

**Figure 1:**
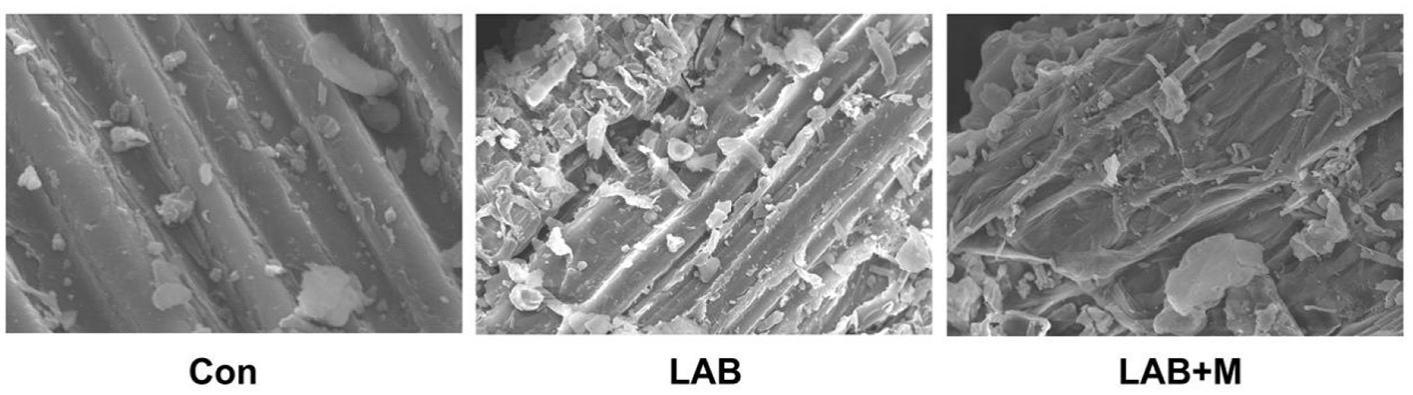
SEM imagines of rice straw residues obtained from treatment with LAB or LAB + M. Con: no additive, control; LAB: added LAB, LAB + M: a combination of LAB and molasses.

The scanning electron microscopy images showed that LAB and LAB + M treatments disrupted the physical structure of rice straw in this study (Figure 1), whereas the morphology of untreated rice straw exhibited a compact, rigid, and angular fibril structure with pilling. In addition, the rice straw treated with both LAB and LAB + M reflected somewhat melted and patchy surfaces.

**Figure 2:**
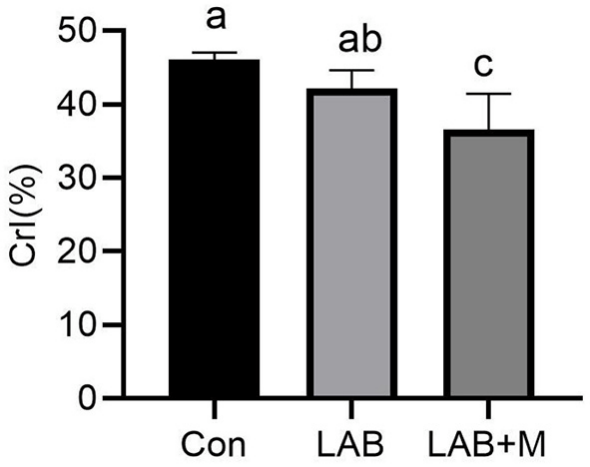
Effect of LAB or LAB + M treatments on the Cellulose crystalline index (CrI, %) of rice straw. Con: no additive, control; LAB: added LAB, LAB + M: a combination of LAB and molasses.

To reveal the changes in cellulose structure, XRD diffraction data were acquired (Figure 2). The CrI of LAB + M was decreased (*p* <0.05) compared to Con.

The NDF (*p* <0.001) and ADF (*p* <0.001) contents (Table 1) were decreased in LAB and LAB + M treatments compared to Con. While the content of DM was lowest in LAB + M (*p* <0.05), no difference was found between LAB and Con (*p* > 0.05). While the content of CP was increased in LAB and LAB + M treatments compared to Con (*p* <0.001), no difference was found between LAB and LAB + M (*p* > 0.05). Meanwhile, after 30 days of ensiling, the NDF (*p* <0.001) and ADF (*p* <0.001) contents of rice straw were lowest in LAB and LAB + M treatments. Additionally, the content of CP was stable at 15 days.

**Table 1:** Effect of LAB or LAB + M treatments and anaerobic storage days on the chemical composition of rice straw.

	**Day**					**Treatments**				***P***		
**Item**	**7**	**15**	**30**	**45**	**SEM**	**Con**	**LAB**	**LAB+M**	**SEM**	**D**	**T**	**D × T**
DM	95.99^d^	96.57^b^	96.76^a^	96.17^c^	0.10	96.49^a^	96.56^a^	96.08^b^	0.11	<0.001	<0.001	0.002
NDF	64.27^a^	60.13^b^	56.52^c^	59.12^b^	0.83	64.11^a^	57.85^b^	58.06^b^	1.07	<0.001	<0.001	0.003
ADF	38.10^a^	36.55^b^	32.77^c^	34.36^c^	0.58	38.79^a^	33.47^b^	34.09^b^	0.89	<0.001	<0.001	0.027
CP	2.93^b^	3.18^a^	3.37^a^	3.19^a^	0.07	3.00^b^	3.26^a^	3.24^a^	0.08	0.002	0.010	0.04

*Different superscript letters a, b, c, and d indicates significantly different values (P <0.05) across-rows, and the same letters indicate insignificant differences (P > 0.05). Con, no additive, control; LAB, added LAB, LAB + M, a combination of LAB and molasses; DM, dry matter (the dry matter content is calculated based on air-drying the sample); CP, crude protein; NDF, neutral detergent fiber; ADF, acid detergent fiber*.

**Figure 3:**
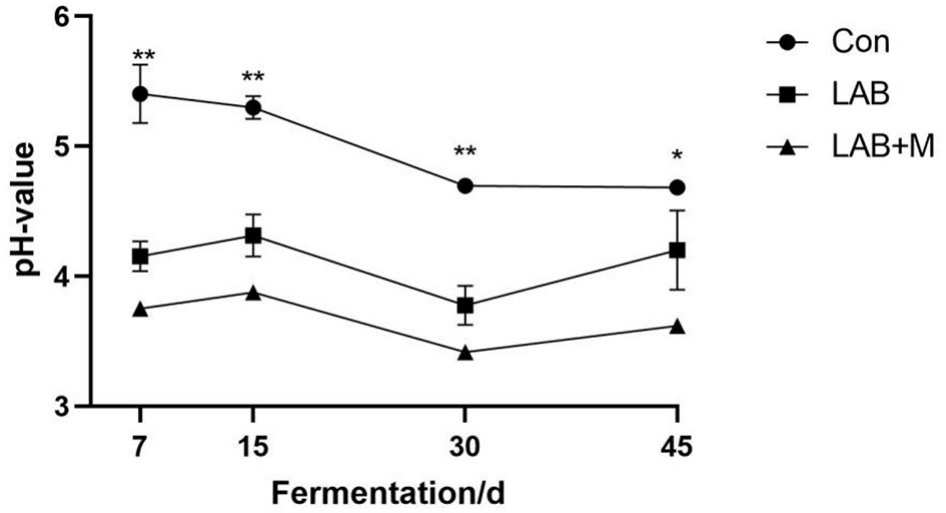
Effect of LAB or LAB + M treatments on the pH value of rice straw. Con: no additive, control; LAB: added LAB, LAB + M: a combination of LAB and molasses. While * and ** indicated the significant correlations at *p* < 0.05 and *p* < 0.01, respectively.

In general, with the extension of the fermentation days, the pH value of each group showed a downward trend (Figure 3). The LAB (3.77) (*p* <0.001) and LAB + M (3.41) (*p* <0.001) treatments were decreased pH value in rice straw after ensiling for 30 days. The LAB and LAB + M treatments were shown lower pH values compared to Con (*p* <0.05) after ensiling for 45 days. Specifically, the pH values were lower 4.0 in LAB + M group.

**Figure 4:**
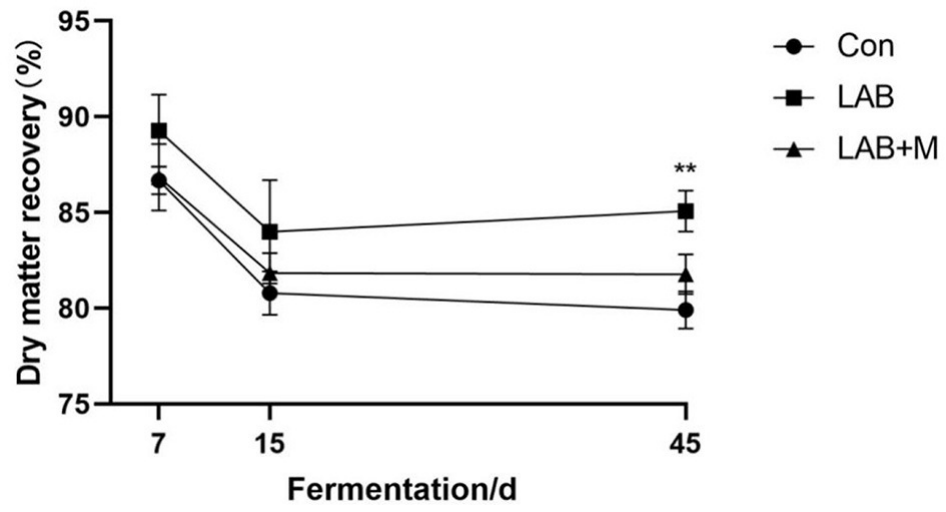
Effect of LAB or LAB + M treatments on the Dry matter recovery (%) of rice straw after anaerobic fermentation 7, 15 and 45 days. Con: no additive, control; LAB: added LAB, LAB + M: a combination of LAB and molasses, while * and ** indicated the significant correlations at *p* < 0.05 and *p* < 0.01, respectively.

As illustrated in Figure 4, dry matter recovery showed a dynamic change during the whole silage period that first decreased and then keep stable. After 45 days, the dry matter recovery was higher in LAB and LAB + M groups (*p* <0.001) in comparison with Con.

### *In vitro* Ruminal Degradation and Total gas Production

The LAB + M and LAB treatments were increased DM (*p* <0.001), NDF (*p* <0.001), and ADF (*p* <0.001) degradation (Table 2) of rice straw compared to Con. The LAB + M was increased gas production (*p* = 0.001) over the 72-h incubation period and asymptotic gas production (*p* = 0.003) compared to LAB and Con, whereas no difference was found between LAB and Con treatments (*p* > 0.05) for gas production and asymptotic gas production.

**Table 2:** Effects of LAB or LAB + M treatments on the biodegradation and gas production after 72 h of *in vitro* ruminal incubation of rice straw.

**Item**	**Con**	**LAB**	**LAB+M**	**SEM**	***P***
Biodegradation (%)					
DM degradation	52.69^c^	59.60^b^	66.02^a^	0.94	<0.001
NDF degradation	49.77^c^	55.20^b^	63.56^a^	1.02	<0.001
ADF degradation	45.86^c^	52.11^b^	60.38^a^	1.09	<0.001
Gas production					
Total gas (mL/g of DM)					
GP_72_	142.99^b^	148.34^b^	176.92^a^	3.46	0.001
A	153.65^b^	155.24^b^	192.48^a^	5.10	0.003
B	1.36	1.54	1.26	0.08	0.117
C	9.54	7.83	8.77	0.55	0.166

*Different superscript letters a, b, and c indicates significantly different values (p <0.05) across rows, and the same letters indicate insignificant differences (p > 0.05). Con: no additive, control; LAB: added LAB, LAB + M: a combination of LAB and molasses. A is the asymptotic gas production (ml/g); B is a sharpness parameter determining the curve's shape; C is the time (h) at which half of A is reached*.

### Particle-Attached Bacterial Density

**Figure 5:**
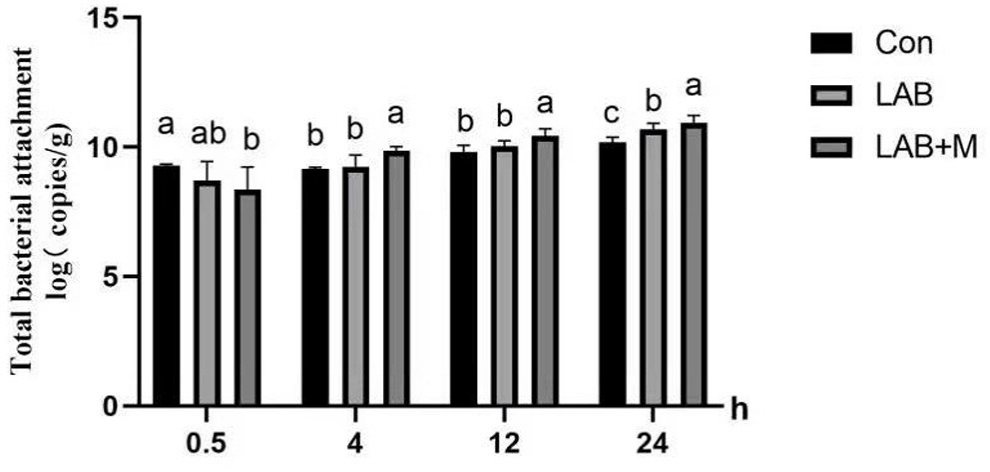
Effect of LAB or LAB + M treatments on rice straw's microbial colonization (MC). Different lowercase letters a, b, and c above the columns indicate significant differences (*p* < 0.05), and the same letters indicate no significant differences (*p* > 0.05). Con: no additive, control; LAB: added LAB, LAB + M: a combination of LAB and molasses.

Total bacterial population in the rice straw samples was estimated by real-time PCR by measuring the total copy number of bacterial 16S rRNA genes (Figure 5). The highest microbial colonization in the samples of rumen-incubated rice straw was observed at 24 h of incubation for LAB +M than LAB and Con treatments. Higher numbers of colonization bacteria (*p* <0.05) were also observed in the LAB + M treatment than Con and LAB groups at 4 to 12 h of incubation. Interestingly, in the Con, the microbial colonization of rice straw surface was the highest at 0.5 h (*p* <0.05) and decreased (*p* <0.05) after 4 h in the rumen.

## Discussion

### Physical Structure and Physicochemical Properties

The degradation difficult of lignocellulose is complex cell wall structure of cellulose–hemicellulose–lignin (24). In this study, the morphology of untreated rice straw exhibited a compact, rigid, and angular fibril structure with pilling, whereas rice straw treated with LAB and LAB + M reflected somewhat melted and patchy surfaces. It indicates that treated rice straw could provide more rumen microbial colonization sites. Notably, the increase in microbial colonization on the surface of rice straw had a positive effect on degradation (25). Furthermore, the relative lower acidic environment may cause the damage to the cell wall structure of rice straw (26). Indeed, the LAB and LAB + M treatments have shown lower pH values in this study (Figure 3); especially, the pH values was lower 4.0 in LAB + M group, whereas the straw surface damage in LAB + M group was the most obvious compared to Con and LAB groups. Also, a study reported that acid pretreatment resulted into solubility of 70–80% of xylan in barley straw (27), which imply that a lower acidic environment could soluble the hemicellulose in forage.

To reveal the changes in cellulose structure, XRD diffraction data were acquired. Unlike starch and hemicellulose, cellulose has a crystalline structure. Its crystallinity is believed to play a major role in its biological conversion. Cellulose CrI is the important parameter on cellulose structural features (28). In this study, the CrI of LAB + M was significantly decreased as expected compared to Con. Notably, the lower CrI could be attributed to the partial hydrolysis of cellulose, reduction in the crystalline material, and increase in the amorphous substances, and suggestion has a positive effect on fiber degradation (29).

The chemical composition in forage is related to forage quality and degradation (30). In this study, the removal of hemicellulose from the cellulose–hemicellulose–lignin structure led to a reduction in NDF and ADF contents in rice straw treated with LAB and LAB + M. Notably, the low NDF and ADF were associated with higher feed quality and degradation, as well as higher dry matter intake (31). Similar results were obtained in rice straw silage, in which LAB + M reduced the NDF and ADF content of rice straw silage (32). This may be due to the enzymes secreted by microbes degrading the crude fiber during fermentation process (33). Meanwhile, after 30 days of ensiling, the NDF and ADF contents were lowest in LAB and LAB + M treatments. In a previous study, ensiling of grass for 7 to 28 days resulted in the degradation of structural carbohydrates by acid hydrolysis at low pH (34). On the other hand, the CP content was significantly increased in the LAB and LAB +M treatments. This might be a suggestion that rice straw treated with LAB and LAB +M rice straw has a positive effect on degradation.

In this study, LAB (3.77) and LAB + M (3.41) treatments decreased the pH value in rice straw after ensiling for 30 days. Notably, the pH values might be used as an indicator to roughly monitor the auto-hydrolysis following pretreatment with feed additives, contributing to the screening of optimum conditions for different kinds of feedstock (35). Moreover, a pH of 4.0 may inhibit microbial activity as well as provide long-term preservation or inhibit downstream biochemical processes (36). Pretreatment biomass losses should be taken into account, which would affect the soluble sugars yield (37). In this study, dry matter recovery showed a dynamic change during the whole silage period that first decreased and then keep stable. This might be attributed to more lignocellulose undergoing hydrolysis, and more volatile products such as organic acids and compounds such as furfural are generated (38). The dry matter recovery is higher in LAB and LAB + M groups than Con after 45 days, and it implies that more nutrition was retained in rice straw after pretreated by LAB or LAB + M.

### *In vitro* Ruminal Degradation and Total gas Production

The release of hemicellulose from the cell wall matrix promotes enzymatic hydrolysis and fermentation of the feed substrate. Neutral detergent soluble, including water-soluble carbohydrates (WSC), proteins, and other extracts, can be degraded easily by microorganisms (39). In this study, LAB and LAB + M treatments increased DM, NDF, and ADF degradation of rice straw, and LAB + M increased the gas production over the 72-h incubation period, asymptotic gas production. Notably, these changes proved that LAB or LAB + M treatments could improve the nutritional value of rice straw.

*In vitro* gas production has been used to predict the rumen degradability and metabolizable energy of different animal feeds (40). A strong correlation between nutritional content and *in vitro* cumulative gas production exists, and these parameters have gained wide acceptance in the nutritional evaluation of animal feeds (41–43). *In vitro* gas production is highly dependent on the availability of soluble fractions, which favor ruminal fermentation at the early fermentation stages (44). In this study, the LAB + M group significantly increased the GP_72_ of rice straw, which suggests that LAB + M treatment could improve the nutritional value of rice straw.

Pretreatment prepares the carbohydrates, particularly cellulose, for an enzymatic or microbial attack. It is well established that CrI is among the parameters that are widely measured and related to the bioconversion of the lignocelluloses (45). Many studies have shown that CrI has a negative effect on digestibility (46, 47). In this study, the LAB + M group significantly reduced the CrI of rice straw, which partially explained the improved digestibility in rice straw.

### Particle-Attached Bacterial Density

Normally, bacteria attached to the feed particles account for about 75% of the total microorganisms in the rumen (48). This indicates that the bacteria attached to the feed particles play a vital role in the digestion and utilization of feed. The highest microbial colonization on the samples of rumen-incubated rice straw was observed at 24 h of incubation, and the LAB + M was highest. Higher microbial colonization was also observed in the LAB + M group than other groups from 4 to 12 h of rumen incubation. The results imply that the degradation of rice straw in the rumen mainly occurs within 24 h. Indeed, the degradation of feed incubated in rumen for 24 h was related to the nutritional value of the feed (49). Interestingly, in control group, the microbial colonization on the rice straw was the highest at 0.5 h. This suggested that the turning point of microbial colonization of low quality pasture was 0.5 h (50).

The rice straw incubated with LAB + M showed the most significant changes in its structure and hence had a larger surface area that allowed for more microbial attachment sites. Unfortunately, we incubated the samples in the rumen for only 24 h. Hence, we cannot independently state whether degradation of rice straw in the rumen could have increased or decreased thereafter. Thus, the LAB or LAB + M pretreatments of rice straw were significantly increased the degradation by ruminal microbiome in this study. The LAB or LAB + M not only destructs the structure of rice straw and provides more rumen microbial colonization sites but also changes the chemical composition, such as decreased NDF, ADF and CrI contents, and increased CP content of rice straw. Importantly, these changes could improve the degradation of rice straw. Indeed, the results of *in vitro* digestion and gas production of rice straw confirmed it. Lactic acid bacteria and molasses in this study may be used to develop large-scale processes to improve the nutritional value of rice straw as forage for ruminants.

## Data Availability Statement

The raw data supporting the conclusions of this article will be made available by the authors, without undue reservation.

## Ethics Statement

The animal study was reviewed and approved by all animal procedures used in the current study were reviewed and approved by the Animal Care Committee of the College of Animal Science and Technology of China Agriculture University (Protocol number: 2013-5-LZ).

## Author Contributions

ZC, XC, and YM mainly designed this experiment. The animal experiment was conducted by XC and YM. Data were collected and analyzed by XC and YM. The manuscript was mainly written by YM and edited by JX, GA, MK, YW, ZC, and SL. All authors contributed to the article and approved the submitted version.

## Funding

This work was funded by the 2115 Talent Development Program of China Agricultural University.

## Conflict of Interest

The authors declare that the research was conducted in the absence of any commercial or financial relationships that could be construed as a potential conflict of interest.

## Publisher's Note

All claims expressed in this article are solely those of the authors and do not necessarily represent those of their affiliated organizations, or those of the publisher, the editors and the reviewers. Any product that may be evaluated in this article, or claim that may be made by its manufacturer, is not guaranteed or endorsed by the publisher.
